# Born during World War II: Associations with lower incident dementia

**DOI:** 10.1002/alz.092127

**Published:** 2025-01-09

**Authors:** Sotiria Moza, Nikolaos Scarmeas, Mary Yannakoulia, Efthimios Dardiotis, Georgios M. Hadjigeorgiou, Paraskevi Sakka, Mary H. Kosmidis

**Affiliations:** ^1^ Lab of Neuropsychology & Behavioral Neuroscience, School of Psychology, Aristotle University of Thessaloniki, Thessaloniki, Thessaloniki Greece; ^2^ Columbia University, New York, NY USA; ^3^ National and Kapodistrian University of Athens, Athens Greece; ^4^ Department of Nutrition and Dietetics, Harokopio University, Athens Greece; ^5^ Department of Neurology, Faculty of Medicine, University of Thessaly, Larissa Greece; ^6^ Department of Neurology, Medical School, University of Cyprus, Nicosia Cyprus; ^7^ Athens Alzheimer Association, Athens, Attica Greece; ^8^ Lab of Neuropsychology & Behavioral Neuroscience, School of Psychology, Aristotle University of Thessaloniki, Thessaloniki Greece

## Abstract

**Background:**

Prior longitudinal studies have found that individuals born during World War II and the postwar period had lower incident dementia (Tom et al., 2020) than previous generations, a finding contradictory to research indicating early‐life stressors as adverse events for late‐life cognition. This study aimed to further explore this association and underlying factors.

**Methods:**

We analysed data from 1063 older Greeks (Dardiotis et al., 2014). Of those, 614 were exposed to WWII during early childhood (≤6 years) (*Mean* ^age^ = 70.61±2.13, *Mean* ^education^ = 8.29 years±4.63, 37.50% men) while 349 were not directly exposed (born after 1944) (*Mean* ^age^ = 66.89±1.37, *Mean* ^education^ = 11.24 years±5.00, 28.70% men). Participants provided sociodemographic characteristics and medical history (number‐type of comorbidities), and were administered neuropsychological tests (standardised scores were grouped into visuospatial, memory, attention/speed, language, and executive function domains), the Mini Mental State Examination (MMSE) and the Clinical Dementia Rating Scale (CDR). Logistic regressions were performed with the two groups and cognitive variables, using sex, age, education, clinical depression and anxiety, and multimorbidity, as covariates. We also performed chi‐squared tests to compare the two groups’ parental occupations, as indices of prior socioeconomic status.

**Results:**

Individuals exposed to WWII in early childhood outperformed those not‐directly‐exposed, in visuospatial tasks and the MMSE, and exhibited lower scores on the CDR scale, even after adjusting for multimorbidity, and clinical depression and anxiety (*Controlling for multimorbidity*: Visuospatial perception: *B* = .538, *p* = .001, *OR*:1.713, *95%CI*:1.236‐2.374, CDR score: *B* = ‐2.012, *p*<.001, *OR*:.134, *95%CI*:.043‐.412, MMSE total score: *B* = .135, *p* = .021, *OR*:1.144, *95%CI*:1.020‐1.283, *Controlling for depression and anxiety*: Visuospatial perception: *B* = .542, *p* = .001, *OR*:1.719, *95%CI*:1.240‐2.383, CDR score: *B* = ‐2.119, *p* = .001, *OR*:.120, *95%CI*:.035‐.409, MMSE total score: *B* = .135, *p* = .021, *OR*:1.145, *95%CI*:1.021‐1.284). Parents in the non‐exposed group were more likely to hold white‐collar jobs than those in the early childhood‐exposed group (mother: *χ^2^* (20) = 41.202, *p* = .004; father: *χ^2^* (24) = 66.959, *p*<.001)

**Conclusions:**

Our results parallel the Tom and colleagues’ study (2020), indicating that individuals exposed to WWII‐related stressors demonstrate superior late‐life cognitive performance relative to non‐exposed individuals, even after accounting for covariates. This would suggest the development of cognitive resilience among those exposed to such traumatic events.

